# The microbiota restrains neurodegenerative microglia in a model of amyotrophic lateral sclerosis

**DOI:** 10.1186/s40168-022-01232-z

**Published:** 2022-03-11

**Authors:** Laura M. Cox, Narghes Calcagno, Christian Gauthier, Charlotte Madore, Oleg Butovsky, Howard L. Weiner

**Affiliations:** 1 Ann Romney Center for Neurologic Diseases, Harvard Medical School, Brigham & Women’s Hospital, Boston, MA 02115 USA; 2 Bordeaux University, INRAE, Bordeaux INP, NutriNeuro, UMR 1286, Bordeaux, France

## Abstract

**Background:**

The gut microbiota can affect neurologic disease by shaping microglia, the primary immune cell in the central nervous system (CNS). While antibiotics improve models of Alzheimer’s disease, Parkinson’s disease, multiple sclerosis, and the C9orf72 model of amyotrophic lateral sclerosis (ALS), antibiotics worsen disease progression the in SOD1^G93A^ model of ALS. In ALS, microglia transition from a homeostatic to a neurodegenerative (MGnD) phenotype and contribute to disease pathogenesis, but whether this switch can be affected by the microbiota has not been investigated.

**Results:**

In this *short report*, we found that a low-dose antibiotic treatment worsened motor function and decreased survival in the SOD1 mice, which is consistent with studies using high-dose antibiotics. We also found that co-housing SOD1 mice with wildtype mice had no effect on disease progression. We investigated changes in the microbiome and found that antibiotics reduced *Akkermansia* and butyrate-producing bacteria, which may be beneficial in ALS, and cohousing had little effect on the microbiome. To investigate changes in CNS resident immune cells, we sorted spinal cord microglia and found that antibiotics downregulated homeostatic genes and increased neurodegenerative disease genes in SOD1 mice. Furthermore, antibiotic-induced changes in microglia preceded changes in motor function, suggesting that this may be contributing to disease progression.

**Conclusions:**

Our findings suggest that the microbiota play a protective role in the SOD1 model of ALS by restraining MGnD microglia, which is opposite to other neurologic disease models, and sheds new light on the importance of disease-specific interactions between microbiota and microglia.

Video abstract

**Supplementary Information:**

The online version contains supplementary material available at 10.1186/s40168-022-01232-z.

## Introduction

Amyotrophic lateral sclerosis (ALS) is a progressive neurodegenerative disorder characterized by the loss of upper and lower motor neurons, leading to muscle weakness, disability, and death, with a median survival of 3 to 5 years [1]. ALS is a genetically and clinically heterogeneous disease in which the interaction between genetic background and environmental factors are thought to play a major role [1]. Familial ALS accounts for approximately 10% of cases, which results from genetic alterations in several genes including superoxide dismutase 1 (SOD1), TAR DNA-binding protein 43 (TDP-43), chromosome 9 open reading frame 72 (C9orf72), and fused in sarcoma (FUS) [2]. The remaining 90% of cases are sporadic, suggesting an important environmental component.

The intestinal microbiota encompasses trillions of organisms that inhabit the gut [3] and can play a role in neurologic diseases by modulating immune responses in the CNS, altering endocrine signaling along the hypothalamic pituitary axis, and by directly signaling through afferent nerves [4]. Studies have found alterations in the gut microbiota of patients with ALS. Brenner et al. studied 25 patients with ALS vs. 32 healthy controls [5] and found alterations in *Ruminococcaceae*. Blacher et al. analyzed a cohort of 37 patients with ALS vs. 29 healthy controls and found decreased abundance of microbial genes involved in nicotinamide and tryptophan metabolism [6]. Nicholson et al. sequenced the largest number of ALS subjects to date (*n* = 68) vs. healthy controls (*n* = 61) and found a decrease in the butyrate producing bacteria, *Roseburia intestinalis* and *Eubacterium rectale* [7]. The changes in butyrate producing bacteria are consistent with a case study in which 5 patients with ALS had low levels of other butyrate-producing bacteria [8].

Depletion of the microbiome has been shown to affect models of neurologic diseases, including mouse models of multiple sclerosis (MS), Parkinson’s disease (PD), Alzheimer’s disease (AD), and the C9orf72 model of ALS, all of which show less disease when treated with antibiotics or when raised under germ free conditions [9–14]. On the other hand, antibiotics were found to worsen survival in SOD1 mice [6] suggesting that the microbiota may play a protective role in the SOD1 model of ALS. In humans, antibiotics have been reported to increase the risk of ALS in a Swedish population [15]. Taken together, these data suggest that alterations in the gut microbiota may affect ALS initiation and progression.

While motor neurons are the primary cell type implicated in the pathophysiology of ALS, it is now clear that ALS arises, in part, through non-cell-autonomous mechanisms that contribute to motor neuron damage. Microglia are resident myeloid cells in the CNS, which become highly dysregulated in ALS and transition from a homeostatic to microglia neurodegenerative (MGnD) phenotype [16]. Patients with ALS show increased proliferation and activation of microglia [17–22] and increased levels of pro-inflammatory cytokines in microglia [23–25]. Modulating levels of mutant SOD1 in motor neurons affects disease onset and the earlier stages of disease [18, 26] whereas selectively silencing mutant SOD1 in microglia delays motor neuron degeneration and extends survival [13]. These studies demonstrate the importance of microglia in disease progression and degeneration of motor neurons in ALS.

It has recently been shown that the gut microbiota can shape microglia both in homeostasis and in disease in models of AD, PD, and the C9orf72 model of ALS [11, 27–29]. To test the role of the microbiota, studies have either used approaches to nearly eliminate the microbiota using high-dose antibiotics or used approaches to modulate the microbiota using low-dose antibiotics. In WT mice, Erny et al. used a combination of high-dose cefoxitin, gentamicin, metronidazole, and vancomycin, all at 1 mg/mL in drinking water (150 mg/kg body weight) and found that the microbiota were necessary to maintain microglia maturation and homeostatic function [27]. In a model of PD, Sampson et al. found that a combination of high-dose ampicillin (1 mg/mL), vancomycin (0.5 mg/mL), neomycin (0.5 mg/mL), gentamycin (0.1 mg/mL), and erythromycin (0.01mg/mL) in the drinking water (1.5–150 mg/kg body weight) improved motor function and decreased microglia activation in alpha-synuclein overexpressing mice [11]. In a model of AD, Minter et al. used a combination of 8 low-dose antibiotics, including gentamycin, vancomycin, metronidazole, neomycin, ampicillin, kanamycin, cefaperazone, colistin [30] (see Table 1 for doses). Antibiotics were initially administered by daily oral gavage for one week at doses between 6.25 and 37.5 mg/kg body weight, followed administration in the drinking water at doses between 1.5 and 9 mg/kg body weight. This treatment was well-tolerated over several months and modulated the gut microbiota without full depletion. This low-dose antibiotic treatment decreased the development of amyloid plaques in male mice [30] and in a follow up study from Dodiya et al., low-dose antibiotics decreased MGnD gene expression in male APP/PS1-21 mice [28]. In the C9orf72 model of ALS, Burberry et al. used high-dose antibiotics delivered by twice daily oral gavage, including ampicillin (200 mg/kg/d), neomycin (200 mg/kg/d), metronidazole (200 mg/kg/d), and vancomycin (100 mg/kg/d) and found that lifelong suppression of the gut microbiota decreased expression of proinflammatory microglial markers and decreased inflammatory and autoimmune phenotypes [29]. Because they observed adverse health effects from long-term high-dose antibiotics, including hepatotoxicity, survival was not investigated in antibiotic treated C9orf72 mice. In the SOD1 model of ALS, Blacher et al. used the same high-dose antibiotic approach as Sampson et al. (metronidazole, ampicillin, neomycin, vancomycin) and found reduced survival [6]. In addition, they also found reduced survival in germ-free SOD1 mice, suggesting that the detrimental effect in SOD1 mice was from the lack of microbes, and not necessarily antibiotic-mediated toxicity. The observation that SOD1 mice have worse disease progression with antibiotics is unique compared to other models of neurologic disease, but whether the gut microbiota affects microglia in SOD1 mice is not known.

**Table 1:** Doses of antibiotics

	Concentration in solution (mg/mL)		Effective dose (mg/kg body weight)	
	Week 1	Week 2 ➔ End	Week 1^a^	Week 2 ➔ End^b^
Gentamicin	1	0.02	12.5	3
Vancomycin	0.5	0.01	6.25	1.5
Metronidazole	2	0.04	25	6
Neomycin	0.5	0.01	6.25	1.5
Ampicillin	1	0.02	12.5	3
Kanamycin	3	0.06	37.5	9
Cefaperazone	1	0.02	12.5	3
Colistin	0.5	0.01	6.25	1.5

^a^Dose calculated based on a 200 μL gavage and average weight of 16 g

^b^Dose calculated based on the average consumption of 0.15 mL water per gram of mouse body weight

We hypothesized that the microbiota influenced disease progression in SOD1 mice by modulating microglia early in the disease course. To test this, we used two different microbiota interventions to either deplete or augment the microbiota. For greater long-term tolerability, we chose to deplete the microbiota using low-dose combination of 8 antibiotics used by Minter et al. [30]. We found that in contrast to studies in models of AD and PD, in the SOD1 mouse, antibiotics promoted an MGnD microglia phenotype and suppressed a homeostatic microglia phenotype early in the disease course, suggesting that one mechanism by which the microbiota may be protective in ALS is by restraining an MGnD phenotype. To augment the microbiota, we co-housed two WT with two SOD1 mice, which is a standard approach to transfer microbiota between two different genotypes [31–34] and has been shown to ameliorate disease in an animal model of AD [31]. Co-housing SOD1 and wild-type littermates did not rescue survival, which may have implications for the ability to ameliorate disease via exposure to microbiota from healthy individuals. These studies further our knowledge of the disease-specific interactions between the microbiota and microglia and sheds light on disease processes that are important to the biology of ALS.

## Materials and methods

### Mice

B6.Cg-Tg(SOD1^G93A^)1Gur/J mice and non-transgenic litter mates were obtained from Jackson Laboratory. SOD1^G93A^ are hemizygous for the G93A mutant form of human *SOD1* and contain high transgene copy number. SOD1 mutation is the second most common mutations found in familial and sporadic ALS, and this model is the most established animal model of the disease. Because our experiments involved cohousing, we utilized female mice to avoid fighting between adult males exposed to new cage mates. All mice were housed under specific pathogen free conditions, with food and water ad libitum. All animals were housed in temperature- and humidity-controlled rooms, maintained on a 12-h/12-h light/dark cycle (lights on at 7:00 AM). Mice were euthanized by CO_2_ inhalation. The Institutional Animal Care and Use Committee (IACUC) at Harvard Medical School and Brigham and Women’s Hospital has all experimental procedures involving animals. For survival experiments, there were 11–13 mice per group. For experiments characterizing microglia at disease onset, there were 4–6 animals per group.

### Antibiotic treatment

SOD^G93A^ mice and non-transgenic littermates were obtained from Jackson Laboratories at 4 weeks of age. Antibiotics were provided at 4 weeks of age by daily oral gavage (200 μL) for 1 week, then provided at a 1/50 dose in drinking water throughout the experiment, including gentamycin 1 mg/mL gavage, 0.02 mg/mL drinking water, vancomycin 0.5 mg/mL gavage, 0.01 mg/mL drinking water, metronidazole 2 mg/mL gavage, 0.04 mg/mL drinking water, neomycin 0.5 mg/mL gavage, 0.01 mg/mL drinking water, ampicillin 1 mg/mL gavage, 0.02 mg/ mL drinking water, kanamycin 3 mg/mL gavage, 0.06 mg/mL drinking water, cefaperazone 1 mg/mL gavage, 0.02 mg/ mL drinking water, colistin 0.5 mg/mL gavage, and 0.01 mg/mL drinking water as previously reported [30]. Antibiotic dose in drinking water and estimated dose in mg/kg body weight in the first week (gavage) and remainder of the experiment (drinking water) are shown in Table 1.

### Cohousing

To augment the microbiota, we co-housed two WT and two SOD1 mice, which is a standard approach to transfer microbiota between two different genotypes. As a control for any stress related to new housing conditions, untreated mice were rehoused with mice of the same genotype to expose them to new cage mates, *n* = 12 per group.

### Behavioral assessment

Clinical assessment of SOD1 mice was conducted by monitoring of body weight, neurological score, and rotarod performance (3 times/week), all starting at day 60 of age. In addition, mice were monitored daily for supportive care, including gel food and water when needed later in the disease course. Disease progression was documented according to established methodology provided by Prize4Life and The Jackson Laboratory. Behavioral assessment was carried out by the same investigator on all groups.

#### Rotarod test

Motor performance was assessed starting at day 60 of age 3 times/week, at the same time of day. On each occasion, the mice were tested three times, with a rest period between runs. The rotarod (Ugo Basile 7650) was set to accelerate from 2 to 40 rpm in 285 s. Rotarod training was performed until all mice reach maximum latency to fall (>285 s). Latency to fall (seconds, s) was recorded in seconds for each mouse. The mean of the three performances, measured as latency to fall in seconds, was used for analysis.

#### Neurological scoring

Neurological score was assessed three times a week for each mouse beginning at 60 days of age. Symptomatic disease onset is defined as the age at which animals presented tremor and/or defective hind limb splay for two consecutive days. The neurological score, using a scale of 0 to 4, was developed by ALSTDI [35, 36]. Criteria used to assign each score level were 0 = full extension of hind legs away from the lateral midline when the mouse is suspended by its tail, and mouse can hold this position for 2 s, suspended 2–3 times; 1 = collapse or partial collapse of leg extension towards lateral midline (weakness) or trembling of hind legs during tail suspension; 2 = curling of the toes and dragging of at least one limb during walking; 3 = rigid paralysis or minimal joint movement, foot not being used for forward motion; and 4 = mouse cannot right itself within 30 s from either side. A score of 4 also corresponded with the humane endpoint of the study.

### Microglia isolation

Microglia isolation was performed according to our previously described protocol [37]. Mice were perfused transcardially with ice-cold Hanks’ Balanced Salt Solution (HBSS), and the spinal cords were hydraulically extruded using an 18 gauge needle and 20-mL syringe of ice-cold HBSS [38]. Single-cell suspensions from the entire spinal cord were prepared using a Dounce homogenizer and centrifuged over a 37%/70% discontinuous Percoll gradient (GE Healthcare) at room temperature. Mononuclear cells were isolated from the interface, washed in Facs buffer, and stained at 4°C for 30 min. To distinguish resident microglia from Ly6C^+^ recruited myeloid cells, we used an anti-LyC6 antibody and a monoclonal antibody that recognizes FCRLS, which is expressed on microglia, but not on infiltrating myeloid cells [16, 37]. Isolated cells were stained with FCRLS-APC (clone 4G11, 3 μg ml^–1^, validated in [37]), CD11b-PeCy7 (clone M1/70, BD Biosciences, 2 μg ml^−1^, validated in reference [37]), and Ly6C antibody [clone HK1.4, Biolegend, 2 μg ml^–1^]. To specifically isolate CNS-resident microglia, we FACS-sorted Ly6C^-^ FCRLS^+^ CD11b^+^ cells.

### RNA sequencing

1000 FACS-sorted CD11b^+^/FCRLS^+^/Ly6C^–^ microglia cells were lysed in 5 ul TCL buffer. cDNA libraries were prepared from sorted cells using the Smart-seq2 protocol [39] by the Broad Technology Labs and sequenced by the Broad Genomics Platform. RNA sequencing was performed using Illumina NextSeq500 using a High Output v2 kit to generate 2 x 25 bp reads. Transcripts were quantified by the BTL computational pipeline using Cuffquant version 2.2.1 [40]. Raw counts were normalized using DESeq2’s (v1.30.1) median of ratios method. Hypothesis testing was performed by using either DESeq2’s Wald test on appropriate variable contrasts or with a likelihood ratio test (LRT) for models with greater than two variable conditions being tested. Adjusted *p*-values were determined using the Benjamini-Hochberg procedure. Volcano plots were generated in R using the EnhancedVolcano (v1.8) package with cutoffs of fold change >0.5 and a *p*-value <0.05. Pathway analysis was conducted using Ingenuity Pathway Analysis [41] for genes with a fold change >0.5 and a *p*-value <0.05. Dot plots were generated in R with ggplot2 and colorBrewer. For comparing genes with altered expression displayed in heatmaps, the cutoff was set at an FDR-adjusted *q* value of 0.2.

### Microbiota sequencing

Fecal samples were collected prior to antibiotic treatment and over the course of the experiment. DNA was extracted using the Qiagen PowerLyzer DNAEasy kit. The V4 16S rRNA gene was amplified with barcoded fusion primers developed by the Earth Microbiome Project [42] and as we have previously described [43]. Libraries were sequenced on the Illumina MiSeq at the Harvard Biopolymers Facility. Downstream analysis was performed in QIIME2 [44], DNA demultiplexing and quality filtering were performed by DADA2, samples were aligned, and alpha and beta-diversity were calculated at a depth of 1000 reads. Taxonomic assignment was made using the Silva database [45]. Statistical analysis was performed using linear discriminant analysis effect size (LEfSe) [46], with the alpha set at 0.05 and the effect size set at 2. LDA scores and *p*-values were plotted in R using the gg plots and ColorBrewer packages. Changes in beta-diversity were determined using the ADONIS test in QIIME2 using the following parameters: treatment, timepoint, cage, strain, and cohort. Pairwise changes in longitudinal changes in beta-diversity were determined in QIIME2 with the with the state column as the timepoint, with state 1 set as day 30 (baseline) and state 2 set as each following time point [47].

## Results

### Antibiotics worsen disease progression in SOD1 mice

**Fig. 1:**
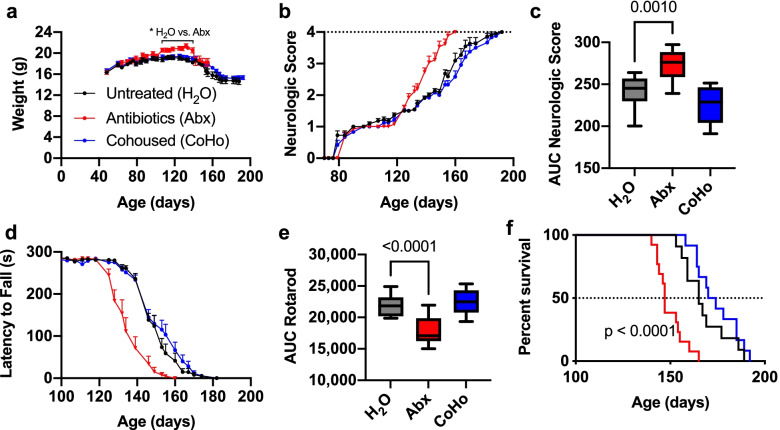
Microbiota depletion worsens disease progression in the SOD1 mouse model. SOD1 mice were treated with low-dose antibiotics (Abx), co-housed with WT mice (CoHo), or untreated (H_2_O), *n* = 11–13 per group. **a** Weight in SOD1 over time. **b** Neurologic score over time shows that ABX mice have a similar age of onset, but faster disease progression compared to untreated H2O, *p* ≤ 0.0001. **c** Area under the curve for cumulative neurologic score. **d** Motor performance by rotarod test over time, *p* ≤ 0.0001. **e** Area under the curve analysis shows that antibiotics worsen cumulative motor performance. Data represent mean ± standard error of the mean (SEM) for **a**–**e**. One-way ANOVA for panels **c** and **e** and two-way ANOVA for panels **a**, **b**, and **d**, with Dunnet’s test for multiple comparison. **F** Survival in SOD1 mice. Dotted line indicates median survival. Mantel–Cox Log-rank test comparison shows reduced survival by 18 days (*p*=0.0002) in SOD1-ABX (median survival 147 days) versus SOD1-H2O (median survival 165 days) mice. SOD1-CoHo did not significantly differ (median survival 172 days) vs. SOD1-H2O

To investigate whether the low-dose antibiotic treatment affects disease progression in the SOD1 animal model of ALS, we administered 8 antibiotics starting at 4 weeks of age by oral gavage daily for 1 week, then administered antibiotics in the drinking water at 1:50 of the dose. This antibiotic treatment regimen is at a lower dose than previously used in SOD1 mice [6] and has been shown to be well tolerated over several months [10]. Antibiotics increased weight in SOD1 mice from day 107 until day 139, then led to a rapid weight loss (Fig. 1a), concurrent with an acceleration in disease progression to a neurologic score of 3. In non-transgenic littermate mice, we also found that antibiotics increased weight (Supplementary Fig. 1a), suggesting that the temporary elevated weight in antibiotic-treated SOD mice reflected the actions of antibiotics on weight, rather than the effect of disease status on weight. This is consistent with our prior work demonstrating that early-life and long-term low-dose antibiotics increase weight gain [48]. The onset of symptoms, as measured by neurologic score, occurred at approximately 80 days of age in both untreated and antibiotic treated SOD mice (Fig. 1b). Antibiotic treatment markedly worsened disease progression, as measured by both increased neurologic score and decreased latency to fall on the rotarod from day 128 until the end of measurement (*p* < 0.05 two-way ANOVA for each timepoint and area under the curve, *p* < 0.001) (Fig. 1b–e). Antibiotics also reduced median survival time by 18 days in SOD1 mice (Fig. 1f). Taken together, these data suggest that dysbiosis from low-dose antibiotics accelerated disease progression and that the microbiome may provide protective factors in the SOD1 model of ALS.

### Cohousing SOD1 mice with non-transgenic littermates does not affect disease progression

Several studies have found that cohousing mice of different genotypes can either transfer disease to WT animals or can ameliorate disease in the genetically susceptible animal. Mice are coprophagic and thus have direct exposure to the microbiota of their cage mates. To determine whether exposure to microbiota from non-transgenic littermates could ameliorate disease, we cohoused two WT mice with two SOD1 mice (CoHo). Untreated mice were rehoused with new cage mates of the same genotype to control for stress and microbiota transmission resulting from novel housing combinations. We did not observe any protective effect of cohousing on neurologic score, motor performance on the rotarod, weight, or survival in SOD1 mice (Fig. 1). Moreover, we did not observe loss of motor function in WT mice cohoused with SOD1 mice (Supplementary Fig. 1). These data indicate that exposure to WT microbiota through cohousing is not sufficient to ameliorate disease in SOD1 mice and that the microbiota from SOD1 mice do not contribute to motor dysfunction in WT animals.

### Antibiotics deplete bacteria that may have beneficial roles in ALS

**Fig. 2:**
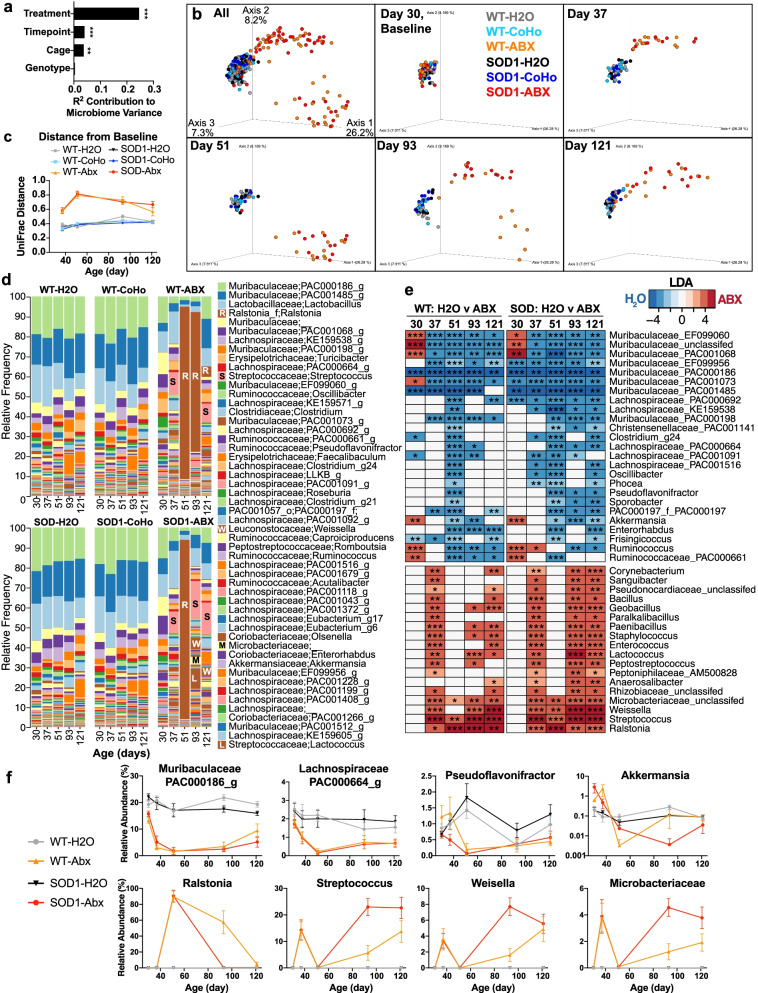
Effect of antibiotics and cohousing on the gut microbiota. SOD1 and WT littermates were treated with antibiotics, cohoused, or did not receive treatment (H_2_O) *n* = 11–13 per group. Microbiota samples were collected at day 30 of life (baseline), then at days 37, 51, and 93, and 121. **a** ADONIS test of microbiota samples. Treatment, timepoint, and cage have a substantial contribution to microbiome variation, whereas strain (WT vs. SOD1) has no effect. ***p=0.001 **b** Principal coordinate analysis of unweighted UniFrac distances show that samples cluster at baseline and are shifted by antibiotic treatment, but not genotype or cohousing. **c** Microbiota divergence from baseline as measured by unweighted UniFrac distances. **d** Microbiota composition over time shows expansion of *Ralstonia*, *Streptococcus*, and *Weisella*. **e** Genera altered by antibiotics that were significant at ≥ 3 timepoints, *p <0.05, **p < 0.01, ***p < 0.001, linear discriminant analysis effect size. **f** Select genera decreased or increased by antibiotic treatment. Statistics shown in panel **e** for LEfSe and in Supplementary Table 1 for pairwise UniFrac distances (**c**)

We next analyzed the gut microbiota to identify bacterial groups depleted by antibiotic treatment and to determine the extent to which the microbiota was transferred between WT and SOD1 mice via cohousing. We sequenced the V4 region of the microbial 16S rRNA gene from fecal samples collected at baseline (day 30), 1 week later (day 37), 3 weeks later (day 51), 2 months later (day 93, early onset), and 3 months later (day 121, early progression). Using the ADONIS test, we investigated the effect of treatment, genotype, cage, and timepoint on overall microbiome variation (Fig. 2a). We found that treatment had the largest effect on microbiota composition, followed by timepoint and cage, but found no differences due to genotype. We next examined the effect of co-housing and antibiotics on beta-diversity at each timepoint in the study (Fig. 2b). All groups clustered together at baseline, after which antibiotic treated mice diverged from untreated and cohoused mice with the greatest changes on day 51 and day 93 of life (Fig. 2c) (longitudinal pairwise UniFrac distance comparisons, Supplemental Table 1). Consistent with changes in beta-diversity, the overall microbiota composition was similar between SOD1 and WT mice. Antibiotics markedly changed populations, whereas cohousing had little effect (Fig. 2d). Antibiotics depleted several Gram-negative anaerobic bacteria, including *Akkermansia* and genera in the *Muribaculaceaeae* family and depleted several butyrate producing members of Class *Clostridia*, including *Clostridium g24*, *Ruminococcus*, and genera in the *Lachnospiraceae* family (Fig. 2e–f)*.* Three weeks following antibiotic treatment, the facultative Gram-negative bacteria *Ralstonia* occupied over 90% of the relative abundance of the gut microbiota, with levels decreasing over time. Antibiotic-treated mice also had increasing levels of antibiotic-resistant bacteria in the class *Bacilli*, including lactic acid producing *Streptococcus* and *Weisella*, two genera known to have both probiotic and pathogenic strains [49–51]. There was also an increase in the antibiotic-resistant *Microbacteriaceae* family. Antibiotic induced changes were similar in both WT and SOD1 mice, suggesting that the combination of genetic risk and microbiota dysbiosis is required for worsening motor function induced by antibiotics.

### Antibiotics amplify the MGnD microglial phenotype in SOD1 mice

**Fig. 3:**
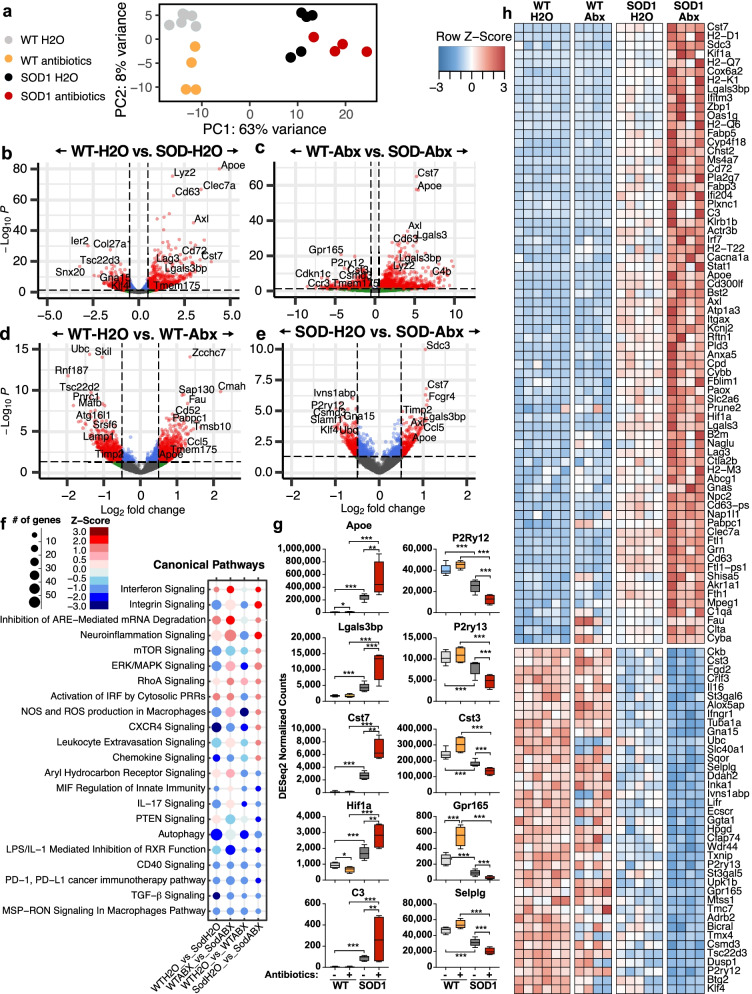
Antibiotics enhance SOD1-associated transcriptional changes in microglia. **a** PCA plot of microglia gene expression. **b**, **c** SOD1 effect: volcano plots of microglia genes that differ between WT and SOD1 mice in H_2_O (**b**) and antibiotic (**c**) treated groups. **d**, **e** Antibiotics effect: volcano plots of microglia genes that differ between H_2_O and antibiotic treated groups in WT (**d**) and SOD1 (**e**) mice. **f** Ingenuity pathway analysis of microglial transcriptional profiles, scaled by number of affected genes (size of circle), and predicted *z*-score for activation (red-blue). **g** DeSeq2 normalized counts of select MGnD genes (left) or homeostatic genes (right) that are amplified by antibiotic treatment. **h** Genes altered in SOD vs. WT mice and further altered by antibiotic treatment in SOD mice, DESeq FDR-adjusted *q* value < 0.2

To investigate the effect of antibiotics on SOD1 microglia in the early stages of disease, we isolated spinal cord microglia at day 120, which corresponds to a time when disease is established but before the antibiotic group showed worsening of disease. This second cohort of mice had similar disease onset, progression, and microbiota changes (Supplemental Fig. 2) as compared to mice used for survival analysis (Figs. 1 and 2). We characterized microglia by RNA sequencing and found distinct transcriptional profiles between SOD1 vs WT mice, based on PCA analysis (Fig. 3a). Microglia from antibiotic treated SOD1 mice were even further from WT mice along PC1, suggesting that the antibiotic treatment exaggerated the genetic effect of SOD1 mutation. As expected, there was a large number of microglia genes altered in SOD1 vs. WT mice in both the untreated (2635 genes) and antibiotic-treated groups (2356 genes) with 1041 genes modulated in the same direction (Fig. 3b, c, Supplemental Fig. 3). Antibiotics modulated 1482 genes in WT mice and modulated 270 genes in SOD1 mice (Fig. 3d, e, Supplemental Figs. 4 and 5). Remarkably, only 26 genes were modulated by antibiotics in both SOD1 and WT mice (Supplemental Fig. 6). Instead, we found that 109 genes that were modulated by antibiotics in SOD1 mice also differed between WT-H2O mice vs. SOD1-H2O mice (Fig. 3b, e, f), suggesting that antibiotics amplified the SOD1-associated microbiota changes.

Specifically, we found that antibiotics in SOD1 mice downregulated homeostatic microglia genes *P2ry12, P2ry13, Selplg, Gpr165*, and *Cst3*. This is consistent with a report from Erny et al. that the microbiota maintains microglia homeostatic function [27]. We also found an increase in genes associated with the microglia MGnD neurodegenerative signature, including apolipoprotein E (*Apoe*), galectin-3 binding protein (*Lgals3bp),* and Cystatin 7 (*Cst7*) [16]. We found an increase in complement factor C3, which can play an important role in synaptic pruning and neuronal death [52]. Furthermore, there was elevated granulin (*Grn*), which has been linked to diseases progression in both ALS patients and SOD1 mice [53]. Elevated Grn may be a compensatory response since overexpression of GRN does not induce motor dysfunction in SOD1 mice and rats [54], and loss of GRN in microglia leads increased inflammatory cytokines and neuronal death [55]. In addition to MGnD genes, Ingenuity Pathway Analysis showed that antibiotic treatment increased pathways involved in neuroinflammation signaling, interferon signaling and inhibition of AU-rich element (ARE)-mediated mRNA degradation, and decreased pathways involved in TGF-β signaling, autophagy, and macrophage-stimulating protein receptor d’origine nantais (MSP-RON) signaling. Of note, these pathways are also modulated in the same direction in SOD-H2O vs. WT-H2O mice, suggesting that antibiotics amplify the SOD1 effect in microglia. There were also pathways that were uniquely modulated, including an increase in production of nitric oxide and reactive oxidative species in macrophages, chemokine signaling, and CXCR4 signaling, suggesting a more inflammatory microglial phenotype in antibiotic treated SOD1 mice.

We compared the effect of antibiotics in both WT and SOD1 mice and found few overlapping genes (Supplemental Fig. 4) compared to unique genes (Supplemental Figs. 5 and 6). Antibiotics modulated 27 genes in the same direction in both WT and SOD1 mice (14 up and 13 down), including upregulation of the MGnD regulator *Apoe* and downregulation of ubiquitin binding protein (*Ubc)*, involved in autophagy. Antibiotics modulated some genes in opposing directions in WT and SOD mice, including *Tmem175*, a lysosomal potassium channel which plays an important role in clearance of autophagosomes [56], and *Gpr165*, a homeostatic microglia gene. These findings indicate that the microbiota may have opposing effects on microglial function in healthy vs. diseased animals.

## Discussion

Our study confirms and extends the finding that the gut microbiota plays a protective role in the SOD1 model of ALS and identifies a new mechanism related to microglia. The microbiota influence over microglia could be mediated by modulating peripheral immune cells that traffic to the CNS or the production of metabolites, which warrants further study. Microglia play an important role in the maintenance of brain homeostasis but lose this homeostatic function in ALS [20, 57]. We recently identified a neurodegenerative molecular signature in microglia from SOD1 mice which we termed MGnD, in contrast to a homeostatic microglial phenotype [16]. Studies have shown that the gut microbiota maintain microglial function in homeostasis and that antibiotics can decrease homeostatic microglia signatures [27]. Consistent with this, we found that antibiotics decreased homeostatic genes *P2ry12, P2ry13*, and *Cst3* in SOD1 mice*.* However, we did not observe a decrease in homeostatic genes in antibiotic treated WT mice, as previously reported in Erny et al. [27]. A key difference is that we used a low-dose antibiotic treatment regimen that modulated the microbiota, whereas Erny et al. used a high-dose antibiotic treatment regimen that mimicked a germ-free state. Unique to our study, we found that antibiotics increased MGnD genes in SOD1 mice, including *Apoe*, *Cst7*, *Lgals*, *Axl*, and others, which is opposite to the effects observed in WT mice and models of AD and PD [11, 27, 28]. We found 109 genes modulated by antibiotics that enhanced SOD1 vs. WT changes in microglia, indicating that an antibiotic-induced dysbiosis amplifies a microglia MGnD phenotype. Furthermore, we found that changes in microglia preceded altered motor dysfunction in antibiotic-treated mice, suggesting that the microbiota slows disease progression by restraining a neurodegenerative microglial phenotype. It is possible that some of these genes, including granulin, reflect early markers of progression and may be a repair response to neurodegeneration. Grn is linked with disease progression in ALS as it is elevated in patients and mice that have progressed in their disease course but is not elevated at disease onset ALS patients compared to healthy controls or pre-symptomatic SOD1 mice [53]. While elevated Grn is associated with ALS, it is suggested to play a protective role by restraining microglia inflammation that leads to neurotoxicty [55].

Because the SOD1 model exhibits a progressive disease that requires more than 4 months of antibiotic intervention, we utilized two microbiota interventions that could lead to a chronic mild depletion (antibiotics) or augmentation (co-housing). In our study, we selected a low-dose antibiotic regimen shown to be well tolerated for long-term administration [10]. This leads to antibiotic-induced dysbiosis, rather than full microbiota depletion. We found that antibiotics initially depleted most populations of the endogenous microbiota, then led to an increase in antibiotic resistant organisms, which may be linked to the altered disease progression that we observe later in the disease course. Antibiotic treatment did not affect the time of disease onset but did affect disease progression, potentially suggesting that administration of antibiotics in the early-symptomatic phase could also have a similar effect of the disease course.

We identified several groups of bacteria depleted by antibiotics that may have beneficial roles in ALS. The Gram-negative anaerobe, *Akkermansia* was depleted at multiple time points. *Akkermansia* has recently been shown to ameliorate disease in antibiotic pre-treated SOD1 mice, which is linked to the production of nicotinomide [58]. *Akkermansia* may also have beneficial roles for other neurologic diseases, including multiple sclerosis [43], epilepsy [59], and Alzheimer’s disease [60]. Antibiotics also depleted several members of *Clostridial clusters IV and XIVa*, which are major butyrate producers in the gut. Two independent studies have found that butyrate producing bacteria were depleted in ALS [7, 8]. Furthermore, administering butyrate to SOD1 mice increased lifespan, increased gut barrier function, and modulated microbiota composition [61], suggesting that of the multiple bacteria targeted by our antibiotic treatment, the disease worsening may be linked to the depletion of butyrate producers. We also found an increase in lactic acid producing bacteria, including *Streptococcus* and *Weissella*, suggesting a shift in metabolic output in antibiotic-treated mice. Both *Streptococcus* and *Weissella* genera have strains that can be either protective or pathogenic [49–51]. Antibiotics also markedly increased the levels of *Ralstonia*, a Gram-negative facultative anaerobe, which is known to carry several antibiotic resistance genes, and can survive in water and cause infection in immunocompromised hosts [62]. Thus, in addition to antibiotics depleting protective bacteria, they may also select for pathogens that can worsen disease.

Recent studies in humans have suggested that antibiotics may contribute to ALS disease onset or progression. In a phase 3 clinical trial, the antibiotic and immunomodulatory agent minocycline worsened ALS [63]. In addition, a study in the Swedish population linked the use of antibiotics to an elevated risk of developing ALS [15]. We did not observe motor dysfunction in antibiotic-treated non-transgenic WT mice. Thus, based on our experimental data, it appears that low-dose antibiotics worsen disease only in genetically susceptibly hosts.

It is possible that antibiotics could have an off-target effect and that antibiotic-mediated toxicity may be responsible for the changes, rather than antibiotic-induced alterations in the gut microbiota. A higher dose combination of antibiotics in the C9orf72 model of ALS initially reduced microglia infiltration into the CNS and ameliorated disease, but then led to off-target health consequences [29]. We did not observe a MGnD microglial phenotype in low-dose antibiotic-treated WT mice. Furthermore, in an animal model of AD, this low-dose combination of 8 antibiotics was well tolerated for several months and reversed a MGnD microglial phenotype [10, 28].

Several studies have found that co-housing a genetically susceptible mouse with a wildtype mouse can transfer disease phenotype, with either the pathogenic or protective trait transferred by co-housing [31–34]. For example, cohousing 5XFAD and WT mice led to cognitive impairment in the WT mice, which was associated with infiltration of Th1 cells in the brain and increased inflammatory cytokines [31]. In our co-housing experiment, we saw no effect on motor function, neurologic score, or survival in SOD1 or WT mice, suggesting that this trait is not transmissible via the microbiota alone. Cohousing may have less of an impact than antibiotics on the microbiome due to inherent colonization resistance. Furthermore, an important point for our study is that we also saw little difference between SOD1 mice and WT littermate microbiota. Other studies have found that differences in the gut microbiota in WT vs SOD1 mice are vivarium dependent [6], and vivarium-dependent changes in the gut microbiota in the C9orf72 ALS animal can determine disease susceptibility [29]. Thus, in a colony of SOD1 mice that have a microbiota distinct from WT mice, cohousing could alter potentially alter disease progression.

## Conclusions

In summary, our study highlights the importance of disease-specific interactions between the microbiota and microglia. Furthermore, given the critical role of microglia in ALS, our finding that the microbiota restrains neurodegenerative microglia in SOD1 mice has important implications for the pathogenesis and treatment of subjects with ALS as detrimental effects of antibiotics have been observed in ALS patients. We identified two groups of bacteria that have been reported to have beneficial roles in ALS, including *Akkermansia* and butyrate-producing bacteria, and further work is needed to confirm their protective role. Finally, we were not able to confer disease protection to SOD1 mice or transmit disease to WT littermates via cohousing, highlighting the importance of the interaction of genetic and environmental risk in the SOD1 model of ALS.

## Supplementary Information

**Additional file 1: Supplemental Figure 1.** Effect of antibiotics on weight in wild-type littermate mice. WT littermate mice were treated with antibiotics (ABX), co-housed with SOD1 mice (CoHo), or untreated (H2O), *n* = 11-12 per group and change in weight and motor function was assessed until SOD1 mice in each treatment group reached humane endpoint criteria (Fig. 1). a) Weight was increased in antibiotic treated mice. Data represent mean ± standard error of the mean (SEM). Multiple unpaired t-tests, adjusted for false-discovery rate, * q< 0.05. b) Neurologic score was assessed along with SOD1 mice. No motor deficits were observed. c) Mice were trained on the rotarod until they could maintain balance for 285 seconds. WT mice were tested along SOD1 mice. No loss in motor function was observed. b-c) data shifted up slightly so that all three groups can be seen.

**Additional file 2: Supplemental Figure 2.** Disease progression and microbiota changes in a second cohort of mice. A second cohort of SOD1 and WT mice were treated with antibiotics, cohoused, or did not receive treatment (H_2_O) *n* = 6 per group. Microglia were then sorted at day 120 of life. a) Neurologic scores were measured 3 times a week, and no difference between treatment groups was observed prior to day 120, which is similar to effects observed in the survival cohort (Fig. 1). b-d) Microbiota samples were collected at day 30 of life (baseline), then at days 37, 51, and 93. b) ADONIS test of microbiota samples from both the survival and microglia sort cohort indicates that treatment and timepoint have a substantial contribution to microbiome variation, whereas the contribution of cage and cohort is small, and genotype has no effect. *** *p* =0.001 c) Principal coordinates analysis of unweighted UniFrac distances show that samples cluster at baseline, and are shifted by antibiotic treatment, but not genotype or cohousing. d) Microbiota composition over time shows expansion of *Ralstonia*, *Streptococcus*, and *Weisella*, which is similar microbiota changes observed in the survival cohort (Fig. 2).

**Additional file 3: Supplemental Figure 3.** The effect of genotype on microglia gene expression in untreated and antibiotic treated SOD1 and WT mice. a) Differential genes (SOD1 vs WT) in both untreated and antibiotic treated mice. DESeq FDR-adjusted q value < 0.2. b) The top 50 upregulated genes up-regulated and down-regulated in both treated and untreated mice. c) DESeq2 normalized levels of selected genes consistently down- or up-regulated by genotype. * *p* < 0.05, ** *p* < 0.01, *** *p* < 0.001.

**Additional file 4: Supplemental Figure 4.** Unique genes modulated by antibiotics in SOD1 mice. A) Differential genes in SOD1-antibiotic (ABX) vs SOD1-H2O that are not altered in SOD1-H2O vs. WT-H2O (Fig. 3). DESeq FDR-adjusted q value < 0.2.

**Additional file 5: Supplemental Figure 5.** Unique genes modulated by antibiotics in WT mice. a) Top 50 genes up and down regulated by antibiotics uniquely in WT mice. DESeq FDR-adjusted q value < 0.2.

**Additional file 6: Supplemental Figure 6.** Genes altered by antibiotics in both SOD1 and WT mice. A-B) Genes modulated by antibiotics in Wt (a) or SOD1 (b). Several genes are regulated in the same direction (upper plot) while others are differentially regulated by antibiotics according to genotype (lower plot). DESeq FDR adjusted q value < 0.2. c) Representative genes modulated by antibiotics.

**Additional file 7: Supplementary Table 1.** Longitudinal comparison of changes in unweighted UniFrac distances.

## Data Availability

The datasets supporting the conclusions of this article are available in the NCBI Short Read Archive data repository, under NCBI Bioproject number PRJNA769453.
