# Supplementary figures and images for: The microbiota restrains neurodegenerative microglia in a model of amyotrophic lateral sclerosis
Source: Microbiome. 2022 Mar 11;10:47. doi: 10.1186/s40168-022-01232-z (PMC8915543; doi:10.1186/s40168-022-01232-z)

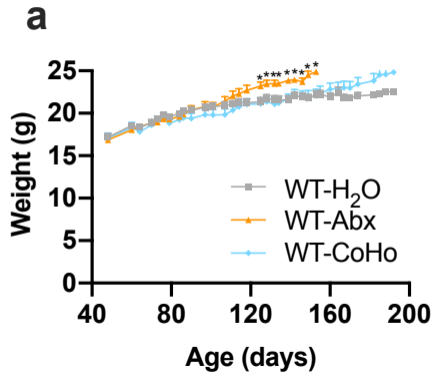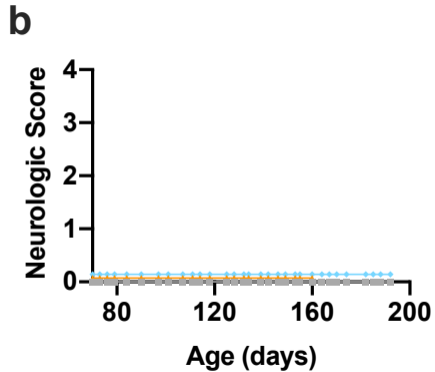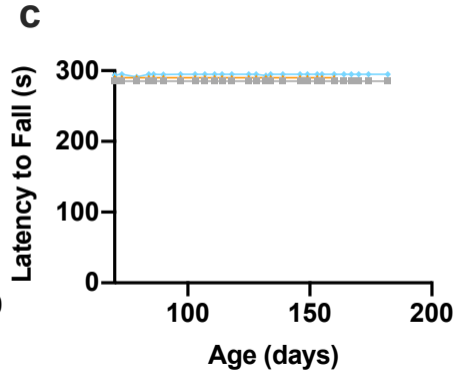

Supplement: Supplementary file 2 — Additional file 1: Supplemental Figure 1. Effect of antibiotics on weight in wild-type littermate mice. WT littermate mice were treated with antibiotics (ABX), co-housed with SOD1 mice (CoHo), or untreated (H2O), n = 11-12 per group and change in weight and motor function was assessed until SOD1 mice in each treatment group reached humane endpoint criteria (Fig. 1). a) Weight was increased in antibiotic treated mice. Data represent mean ± standard error of the mean (SEM). Multiple unpaired t-tests, adjusted for false-discovery rate, * q< 0.05. b) Neurologic score was assessed along with SOD1 mice. No motor deficits were observed. c) Mice were trained on the rotarod until they could maintain balance for 285 seconds. WT mice were tested along SOD1 mice. No loss in motor function was observed. b-c) data shifted up slightly so that all three groups can be seen. [file 40168_2022_1232_MOESM2_ESM.pdf]

**a** Sort Cohort

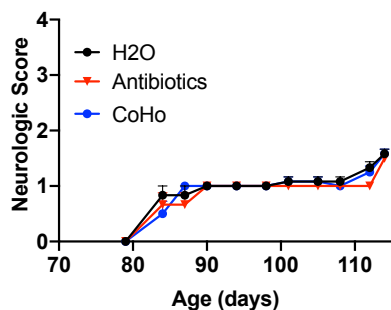

**b** ADONIS test of Unweighted UniFrac

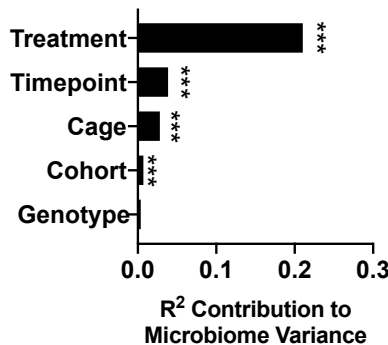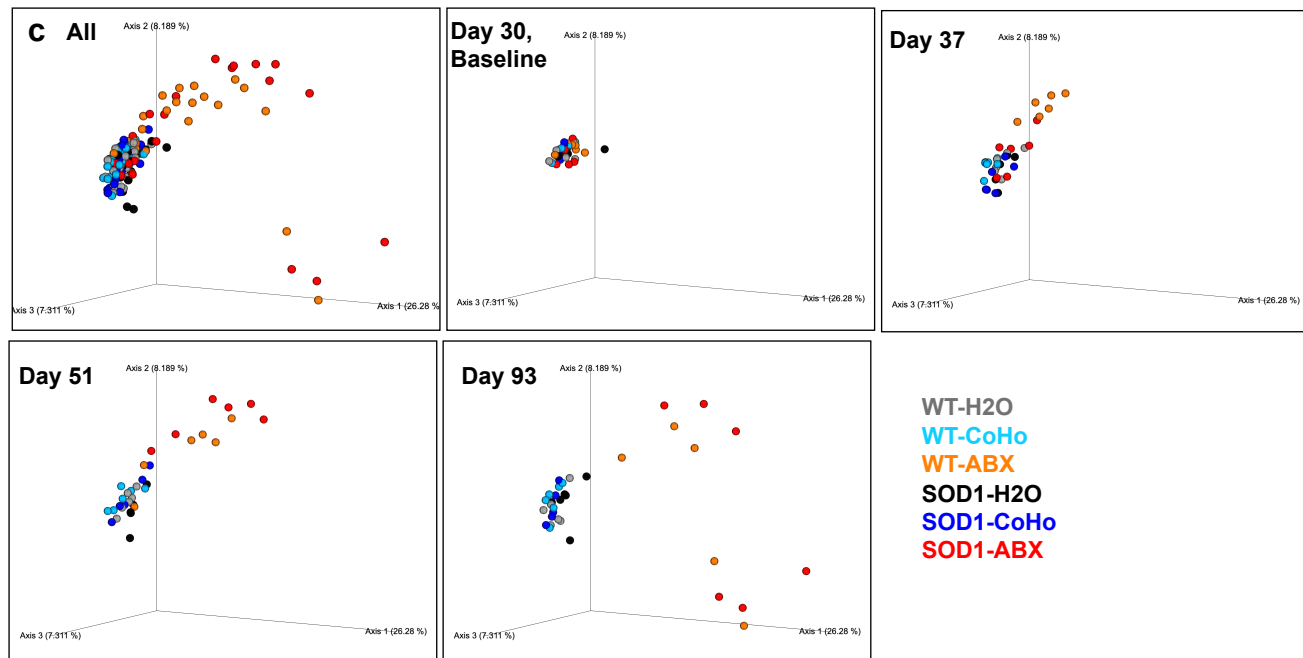

**d**

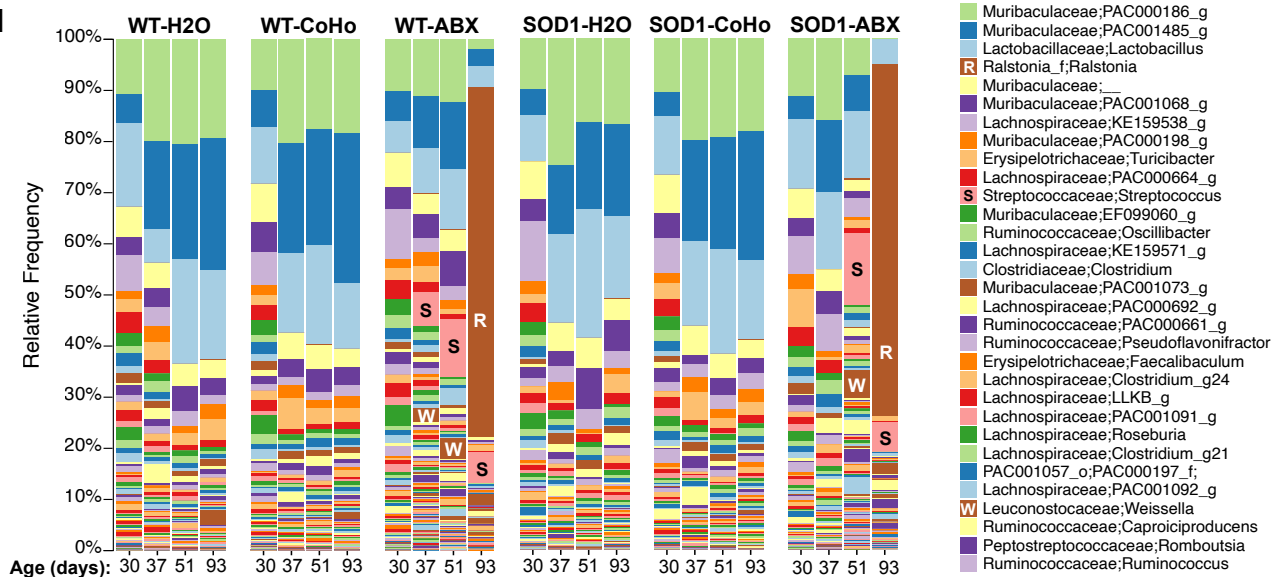

Supplement: Supplementary file 3 — Additional file 2: Supplemental Figure 2. Disease progression and microbiota changes in a second cohort of mice. A second cohort of SOD1 and WT mice were treated with antibiotics, cohoused, or did not receive treatment (H2O) n = 6 per group. Microglia were then sorted at day 120 of life. a) Neurologic scores were measured 3 times a week, and no difference between treatment groups was observed prior to day 120, which is similar to effects observed in the survival cohort (Fig. 1). b-d) Microbiota samples were collected at day 30 of life (baseline), then at days 37, 51, and 93. b) ADONIS test of microbiota samples from both the survival and microglia sort cohort indicates that treatment and timepoint have a substantial contribution to microbiome variation, whereas the contribution of cage and cohort is small, and genotype has no effect. *** p =0.001 c) Principal coordinates analysis of unweighted UniFrac distances show that samples cluster at baseline, and are shifted by antibiotic treatment, but not genotype or cohousing. d) Microbiota composition over time shows expansion of Ralstonia, Streptococcus, and Weisella, which is similar microbiota changes observed in the survival cohort (Fig. 2). [file 40168_2022_1232_MOESM3_ESM.pdf]

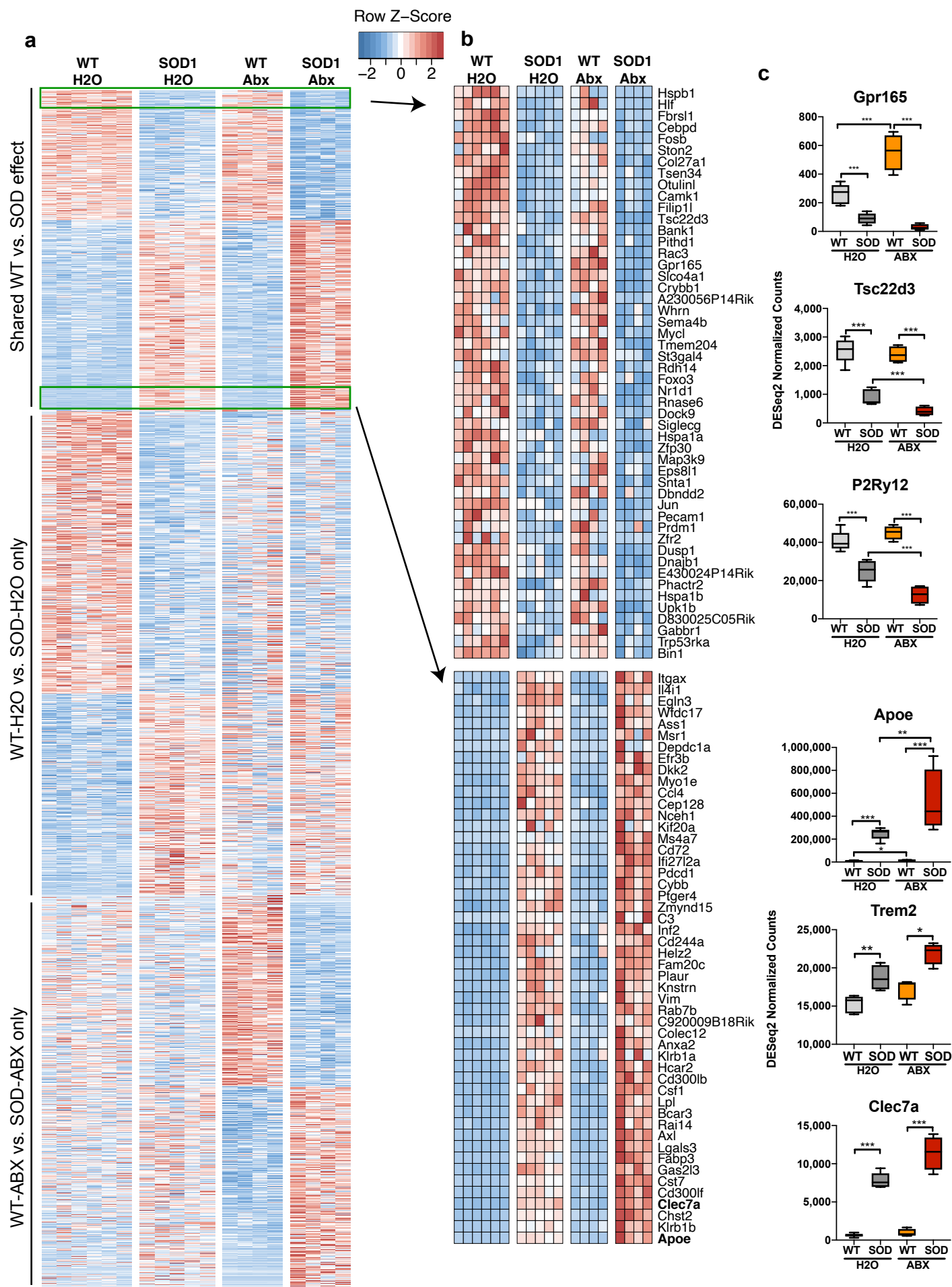

Supplement: Supplementary file 4 — Additional file 3: Supplemental Figure 3. The effect of genotype on microglia gene expression in untreated and antibiotic treated SOD1 and WT mice. a) Differential genes (SOD1 vs WT) in both untreated and antibiotic treated mice. DESeq FDR-adjusted q value < 0.2. b) The top 50 upregulated genes up-regulated and down-regulated in both treated and untreated mice. c) DESeq2 normalized levels of selected genes consistently down- or up-regulated by genotype. * p < 0.05, ** p < 0.01, *** p < 0.001. [file 40168_2022_1232_MOESM4_ESM.pdf]

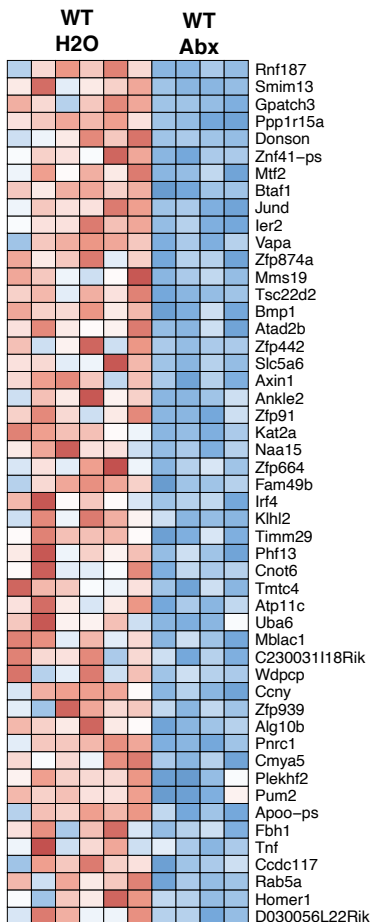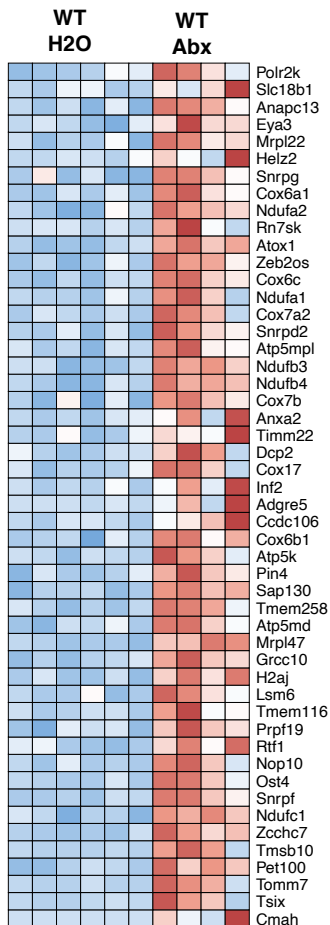

Supplement: Supplementary file 6 — Additional file 5: Supplemental Figure 5. Unique genes modulated by antibiotics in WT mice. a) Top 50 genes up and down regulated by antibiotics uniquely in WT mice. DESeq FDR-adjusted q value < 0.2. [file 40168_2022_1232_MOESM6_ESM.pdf]

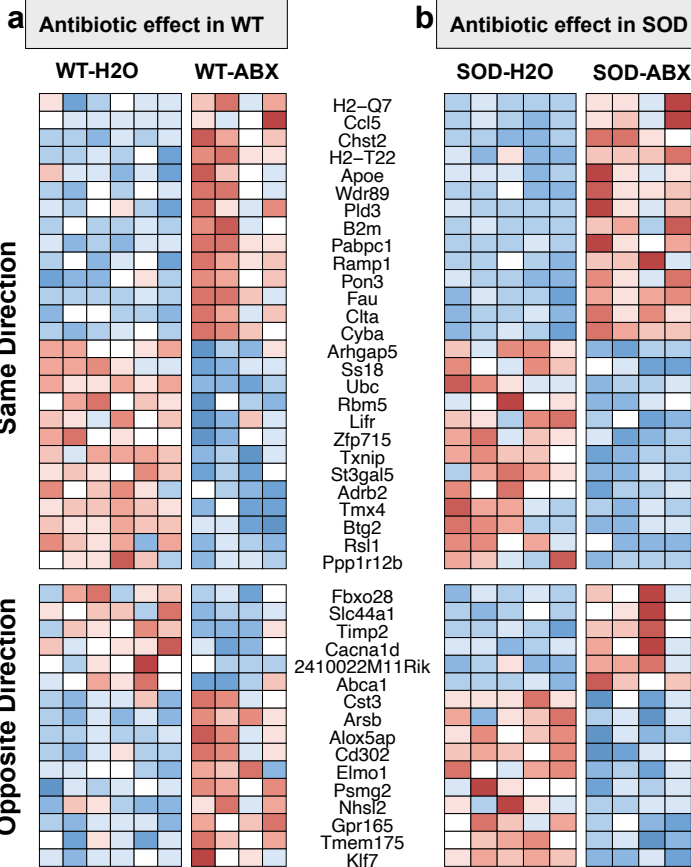

Row Z-Score

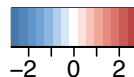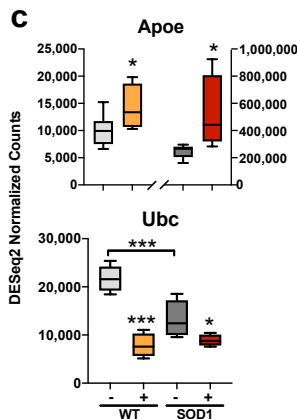

Supplement: Supplementary file 7 — Additional file 6: Supplemental Figure 6. Genes altered by antibiotics in both SOD1 and WT mice. A-B) Genes modulated by antibiotics in Wt (a) or SOD1 (b). Several genes are regulated in the same direction (upper plot) while others are differentially regulated by antibiotics according to genotype (lower plot). DESeq FDR adjusted q value < 0.2. c) Representative genes modulated by antibiotics. [file 40168_2022_1232_MOESM7_ESM.pdf]
