# Supplementary material for: The microbiota restrains neurodegenerative microglia in a model of amyotrophic lateral sclerosis
Source: Microbiome. 2022 Mar 11;10:47. doi: 10.1186/s40168-022-01232-z (PMC8915543; doi:10.1186/s40168-022-01232-z)
Supplement: Supplementary file 5 — Additional file 4: Supplemental Figure 4. Unique genes modulated by antibiotics in SOD1 mice. A) Differential genes in SOD1-antibiotic (ABX) vs SOD1-H2O that are not altered in SOD1-H2O vs. WT-H2O (Fig. 3). DESeq FDR-adjusted q value < 0.2. [file 40168_2022_1232_MOESM5_ESM.pdf]

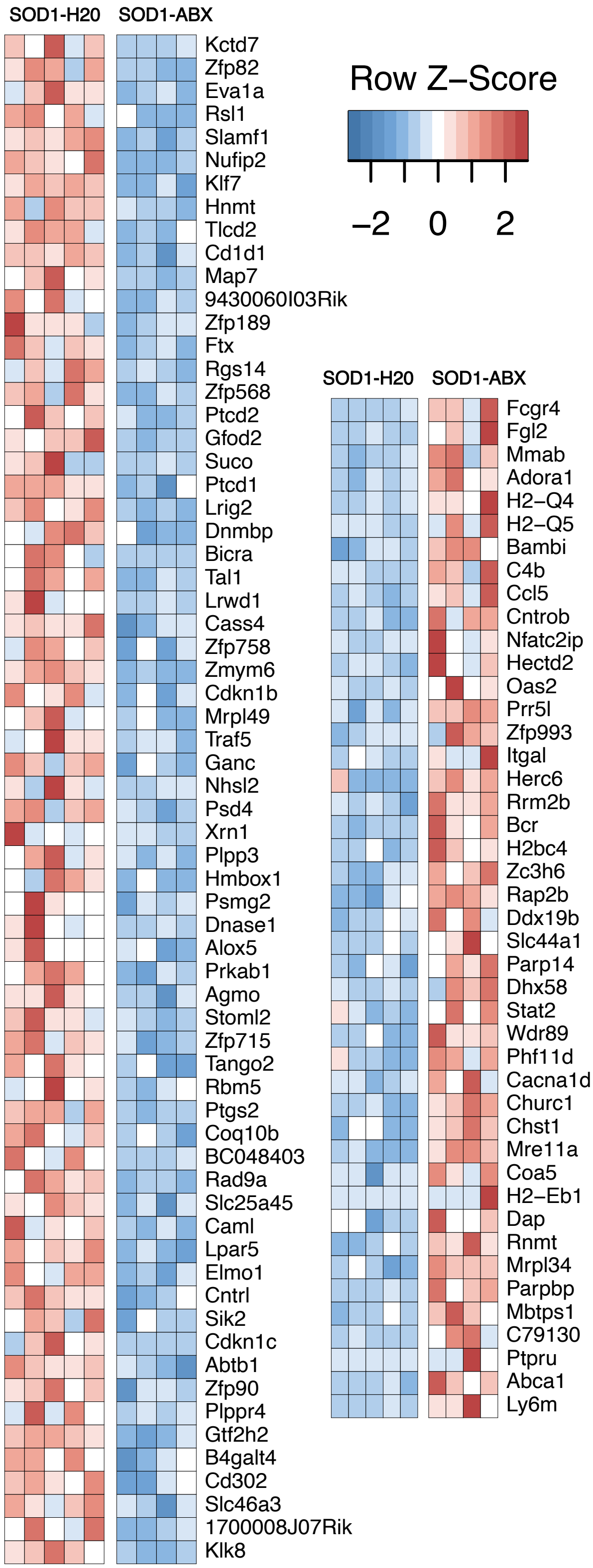
SOD1-H20
SOD1-ABX
Kctd7
Zfp82
Eva1a
Rsl1
Slamf1
Nufip2
Klf7
Hnmt
Tlcd2
Cd1d1
Map7
9430060I03Rik
Zfp189
Ftx
Rgs14
Zfp568
Ptcd2
Gfod2
Suco
Ptcd1
Lrig2
Dnmbp
Bicra
Tal1
Lrwd1
Cass4
Zfp758
Zmym6
Cdkn1b
Mrpl49
Traf5
Ganc
Nhsl2
Psd4
Xrn1
Plpp3
Hmbox1
Psmg2
Dnase1
Alox5
Prkab1
Agmo
Stoml2
Zfp715
Tango2
Rbm5
Ptgs2
Coq10b
BC048403
Rad9a
Slc25a45
Caml
Lpar5
Elmo1
Cntrl
Sik2
Cdkn1c
Abtb1
Zfp90
Plppr4
Gtf2h2
B4galt4
Cd302
Slc46a3
1700008J07Rik
Klk8
Row Z–Score
−2
0
2
SOD1-H20
SOD1-ABX
Fcgr4
Fgl2
Mmab
Adora1
H2–Q4
H2–Q5
Bambi
C4b
Ccl5
Cntrob
Nfatc2ip
Hectd2
Oas2
Prr5l
Zfp993
Itgal
Herc6
Rrm2b
Bcr
H2bc4
Zc3h6
Rap2b
Ddx19b
Slc44a1
Parp14
Dhx58
Stat2
Wdr89
Phf11d
Cacna1d
Churc1
Chst1
Mre11a
Coa5
H2–Eb1
Dap
Rnmt
Mrpl34
Parpbp
Mbtps1
C79130
Ptpru
Abca1
Ly6m
