# Supplementary material for: The microbiota restrains neurodegenerative microglia in a model of amyotrophic lateral sclerosis
Source: Microbiome. 2022 Mar 11;10:47. doi: 10.1186/s40168-022-01232-z (PMC8915543; doi:10.1186/s40168-022-01232-z)
Supplement: Supplementary file 8 — Additional file 7: Supplementary Table 1. Longitudinal comparison of changes in unweighted UniFrac distances. [file 40168_2022_1232_MOESM8_ESM.docx]

**Supplementary Table 1. Longitudinal comparison of changes in unweighted UniFrac distances.**

|  | **Comparison** | **Day 30 🡪 37** | **Day 30 🡪 Day 51** | **Day 30 🡪 93** | **Day 30 🡪 121** |
| --- | --- | --- | --- | --- | --- |
| **Genotype Effect** | WT.H2O vs SOD1.H2O | **0.03024** | **0.04884** | **0.03024** | 0.69359 |
|  | WT.CoHo vs SOD1.CoHo | 0.66501 | 0.62360 | 0.66501 | 0.66501 |
|  | WT.ABX vs SOD1.ABX | 0.37084 | 0.75832 | 0.37084 | 0.18266 |
| **Antibiotic Effect** | WT.ABX vs WT.H2O | **0.00511** | **0.00008** | **0.00511** | 0.09055 |
|  | SOD1.ABX vs SOD1.H2O | **0.00064** | **0.00004** | **0.00064** | **0.00214** |
| **CoHo Effect** | WT.CoHo vs WT.H2O | **0.02100** | 0.10290 | **0.02100** | 0.40605 |
|  | SOD1.CoHo vs SOD1.H2O | 0.73499 | 0.55876 | 0.73499 | 0.87772 |
